# AltaValve Atrial Fixation System for the Treatment of Severe Mitral Regurgitation and Mitral Annular Calcification

**DOI:** 10.1016/j.shj.2024.100294

**Published:** 2024-03-15

**Authors:** Philippe Généreux, Krzysztof Wróbel, Michael J. Rinaldi, Thomas Modine, Vinayak Bapat, Vlasis Ninios, Paul Sorajja

**Affiliations:** a Gagnon Cardiovascular Institute at Morristown Medical Center, Morristown, New Jersey, USA; b Warsaw Medicover Hospital, Warsaw, Poland; c Lazarski University, Warsaw, Poland; d Atrium Health Sanger Heart and Vascular Institute, Charlotte, North Carolina, USA; e Bordeaux University Hospital, Bordeaux, France; f Minneapolis Heart Institute, Minneapolis, Minnesota, USA; g Interbalkan Medical Center, Thessaloniki, Greece

**Keywords:** Mitral annular calcification, Mitral regurgitation, Transcatheter mitral valve replacement

## Abstract

**Background:**

Treatment options for patients with mitral regurgitation (MR) and mitral annular calcification (MAC) are limited. The limitations of current transcatheter mitral valve replacement (TMVR) technologies include high screen failure rates, increased risk of left ventricular outflow tract obstruction, and high residual regurgitation. The aim of this study was to evaluate outcomes of TMVR with the AltaValve system (4C Medical, Maple Grove, MN), a supra-annular TMVR with atrial fixation, in patients with severe MR and moderate or severe MAC.

**Methods:**

Six patients with moderate or severe MAC who were treated with AltaValve TMVR had procedural and mid-term outcomes available.

**Results:**

Technical success was achieved in all patients. Median follow-up was 232 days. At discharge, 80% of patients had none/trace MR, and 20% had mild MR. There was no intraprocedural mortality, device malposition, embolization, or thrombosis. One patient expired 3 days postprocedure due to complications related to the transapical access. All other patients were discharged from the hospital without issues. Echocardiography assessments at 30 days showed complete resolution of MR in all patients, with 1 patient with mild MR and a mean mitral valve gradient of 3.7 ± 1.4 mmHg. All patients were in New York Heart Association Class I/II at 30-day follow-up, showing marked improvement as compared with baseline.

**Conclusions:**

In patients with severe MR and severe MAC, the AltaValve TMVR technology may represent a viable treatment option. The atrial fixation minimizes the risk of left ventricular outflow tract obstruction and potentially expands treatable patients, especially in patients with MAC.

## Highlights

• Atrial fixation of the AltaValve minimizes the risk of left ventricular outflow tract obstruction.
• AltaValve can be delivered transeptally or transapically.
• AltaValve demonstrated positive procedural outcomes in 6 patients with severe mitral annular calcification.
• AltaValve demonstrated resolution of mitral regurgitation in all patients with improved hemodynamics.

## Introduction

Mitral annular calcification (MAC) is estimated to occur in up to 23% of patients presenting with either mitral stenosis or mitral regurgitation (MR), and its prevalence increases with age.^1^, ^2^, ^3^ Patients with MAC are at increased risk for cardiovascular morbidity and mortality.^4^, ^5^, ^6^ The presence of MAC has also been associated with an increased risk of stroke, arrhythmias, heart failure, and perioperative complications.^4^^,^^7^ Currently, treatment options for patients with MAC are not only limited but also complicated in their approach. Surgical treatment may represent a viable option in some patients; however, the presence of MAC increases surgical complexity and is known to lead to a 6-fold increase in operative mortality.^5^^,^^8^

In high and prohibitive surgical-risk patients, transcatheter mitral valve replacement (TMVR) may provide a less invasive treatment option. Major limitations of currently available TMVR procedures include high screen failure rates (reported >40%), the increased risk of left ventricular outflow tract (LVOT) obstruction, and high residual MR.^9^ These risks often require mitigating ancillary procedures associated with procedural complexity and risk.^8^^,^^9^

Recently, the use of an atrial fixation TMVR technology (AltaValve, 4C Medical Technologies) to treat patients at risk of LVOT obstruction has been reported.^10^^,^^11^ Three patients were successfully treated using a transseptal approach and delivery system with good clinical outcomes. The AltaValve is oversized to the left atrium for anchoring and marginally anchored to the mitral annulus for sealing. Since it is not rigidly anchored to the native mitral valve, it may be beneficial in patients with moderate or severe MAC. In this study, we report the use and clinical outcomes for the treatment of six consecutive patients diagnosed with MR and moderate or severe MAC using the AltaValve TMVR technology.

## Methods

### Study Design and Patients

All patients were treated either on a compassionate use basis or enrolled as part of the AltaValve Early Feasibility Study (EFS) (NCT03997305). These patients were recruited at 5 different sites globally with 3 sites in the US (Morristown Medical Center, Morristown, NJ; Abbott Northwestern Hospital, Minneapolis, MN; Atrium Health, Charlotte, NC) and 2 sites in the European Union (Medicover Hospital, Warsaw, Poland; Centre Hospitalier Universitaire Bordeaux, Bordeaux, France). All patients (1) presented with symptomatic 3+ or 4+ MR; (2) were determined to be at high or prohibitive surgical risk by the local heart team; (3) had moderate or severe MAC; and (4) had symptoms of heart failure (New York Heart Association [NYHA] class > II). Exclusion criteria for patients included severe left ventricular (LV) dysfunction (ejection fraction <30%), severe pulmonary hypertension (systolic pressure >70 mm Hg), advanced kidney dysfunction (estimated glomerular filtration rate <30 mL/min), and severe tricuspid regurgitation.

Of these 6 patients, 5 were treated as part of the EFS clinical trial, and 1 patient was treated under compassionate use considerations. Appropriate hospital ethics committee approval, regulatory approval, and informed consent were obtained from all patients prior to the procedure.

### Device Overview

**Figure 1:**
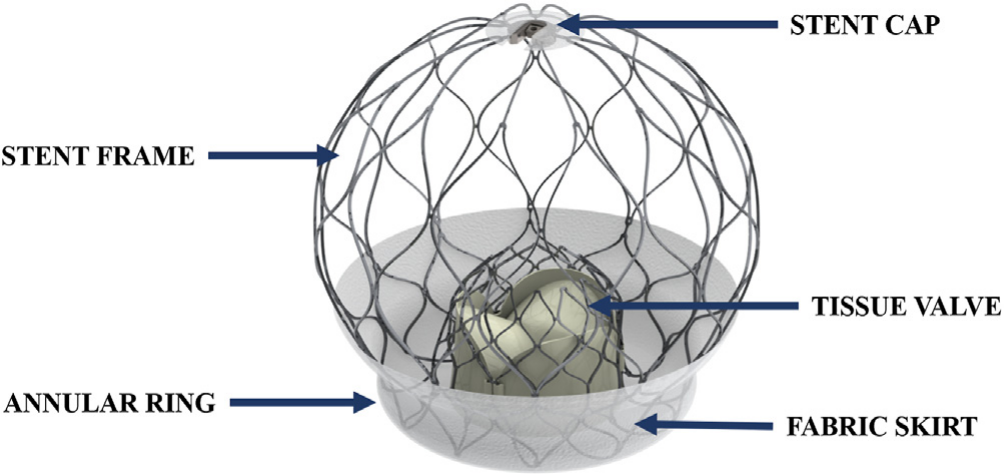
**AltaValve bioprosthesis overview.** The AltaValve consists of a nitinol self-expanding stent frame that houses a 27-mm tri-leaflet bovine pericardium tissue. The device is attached to the delivery catheter via the stent cap, which allows for device deployment and recapture, if needed. The annular ring is covered in fabric to promote sealing against the native mitral annulus. It can be implanted transeptally or transapically.

The AltaValve (Figure 1) is composed of a self-expanding nitinol stent frame that houses a 27-mm tri-leaflet bovine pericardial valve. The outer frame is oversized to the dimensions of the left atrium for anchoring. The ventricular end of the implant, termed the annular ring, is covered by a fabric skirt for sealing and to minimize paravalvular leak (PVL). The annular ring is oversized to the dimensions of the native mitral valve annulus and is available in 3 sizes (40, 46, and 54 mm).

Preprocedural planning is completed using computed tomography (CT) and echocardiography analysis. The AltaValve is delivered via trans-apical (34F) or transfemoral (29F) access. The implant may be delivered without rapid ventricular pacing. Predilation of the mitral valve annulus using a valvuloplasty balloon was not required or attempted in any of the cases. Postprocedure, a 6-month anticoagulation regimen (warfarin or non-vitamin K oral anticoagulants) was recommended for all patients.

### Anatomic Analysis

**Figure 2:**
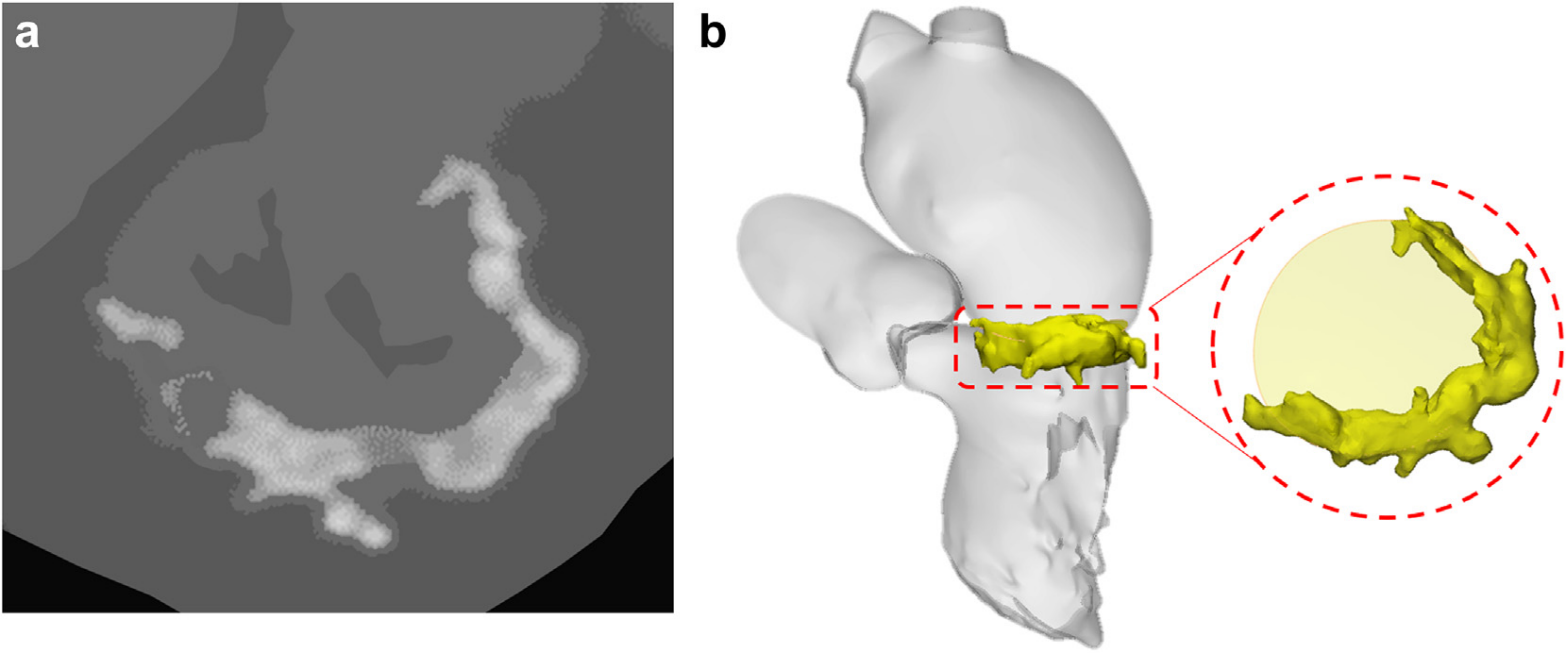
**Mitral annular calcium evaluation.** (a) The Hounsfield threshold value that isolated mitral annular calcification (MAC) was used to create a 3-dimensional model (b) of the MAC. Descriptive characteristics of MAC such as volume, distribution, and thickness were then calculated from this 3-dimensional model.

Anatomic assessment was completed using gated, contrast-enhanced CT images obtained during a complete cardiac cycle. All CT analysis was completed using Mimics Medical (Materialise Inc, Belgium) for the sizing of the left atrium and the mitral annulus. Dimensions of the left atrium were measured in systole for AltaValve sizing. All patients met anatomical inclusion criteria including (1) dimensions of the left atrium <85 mm in systole; (2) dimensions of the native mitral annulus >29 mm and <51 mm in diameter; and (3) volume change of the left atrium <20% between systole and diastole. The severity of MAC was assessed using two methods: total volume >750 mm^3^ and the methodology described by Guerrero et al.^12^ based on the volume and distribution of MAC within the anatomy. Specifically, Guerrero et al. quantified four characteristics of MAC to assess its severity: thickness, degree of distribution, involvement of MAC at the trigones, and involvement of MAC in the valve leaflets. These factors are evaluated using a prescribed scale and then converted to a numerical score to calculate the MAC severity. For each subject, a Hounsfield threshold value that isolated MAC (Figure 2a) was used to create a 3-dimensional model (Figure 2b) of the MAC for the description of severity. All echocardiography data for the EFS patients were analyzed by an independent core lab (Baim Institute for Clinical Research, Boston, MA).

### Clinical Evaluation and Outcomes

The primary safety endpoints assessed include (1) technical success measured at exit from the procedure room and (2) all-cause mortality at 30 days, 3 months, and 1 year thereafter. Technical success at completion of the procedure is defined as (1) absence of procedural mortality; (2) successful delivery and deployment of the implant; (3) successful retrieval of the delivery system; and (4) freedom from emergency surgery or reintervention related to the implant. The primary device performance assessment includes (1) absence of MR grade ≥2+ and (2) valve gradient ≤10 mmHg postprocedure. Follow-up data and images from the treated compassionate use patient were provided by the site to the sponsor. Secondary end points assessed for all patients include clinical adverse events including stroke, bleeding, myocardial infarction, hemolysis, endocarditis, need for reintervention, recurrent heart failure hospitalization, device embolization, LVOT obstruction, and electrical conduction issues. Echocardiographic data were assessed at an independent core laboratory using standard quantitative and qualitative MR assessment.

## Results

### Baseline Demographics

Six consecutive patients with moderate or severe MAC were treated in the study with 4 (67%) of the patients being female. Three (50%) patients were treated using a transapical approach and delivery, and three patients were treated using a transseptal approach and delivery. The mean age was 81.8 years. All patients were diagnosed with at least 3+ MR with a mean LV ejection fraction of 57% (min 44%, max 66%). Of these patients, 5 (83%) presented with advanced heart failure and NYHA class of III or IV. Atrial fibrillation, either paroxysmal or persistent/permanent, was present in 5 (83%) patients. Baseline patient characteristics are presented in Table 1.

**Table 1:** Baseline patient characteristics

Parameter	N = 6
Age, y	81.8 ± 4.1
Female sex	67% (4)
Body mass index, kg/m^2^	28.4 ± 4.1
STS-PROM, %	5.4 ± 2.5
NYHA class	
II	17% (1)
III/IV	83% (5)
LVEF, %	56.8 ± 10.0
HFpEF (≥55%)	67% (4)
HFrEF (<55%)	33% (2)
History of CAD∗	50% (3)
MR severity	
Moderate to severe (3+)	17% (1)
Severe (4+)	83% (5)
MR etiology	
Functional MR	50% (3)
Degenerative MR	33% (2)
Mixed MR	17% (1)
Atrial fibrillation	
None	17% (1)
Paroxysmal	83% (5)
Persistent	0% (0)
Glomerular filtration rate <60 mL/min	67% (4)
Prior valve intervention or surgery†	17% (1)
Prior coronary artery bypass grafting	0% (0)

*Notes.* Values are presented as mean ± SD or % (n).

Abbreviations: CAD, coronary artery disease; HFpEF, heart failure with preserved ejection fraction; HFrEF, heart failure with reduced ejection fraction; LVEF, left ventricular ejection fraction; MR, mitral regurgitation; NYHA, New York Heart Association; STS-PROM, Society of Thoracic Surgeons Predicted Risk of Mortality.

∗ Defined as prior percutaneous coronary intervention, prior coronary artery bypass grafting, or lesion of >50% on coronary angiogram.

† Includes prior mitral repair or replacement, aortic replacement, or tricuspid repair or replacement.

### MAC Description

Based on CT, the average volume of MAC was 2043 ± 916 mm^3^ (Table 2). The mitral annulus diameters ranged from 31 to 51 mm. Based on the descriptive scoring system provided by Guerrero et al., 50% of patients treated were scored to have moderate MAC, with the remainder of the patients having severe MAC (Table 3).

**Table 2:** Mitral valve dimensions and mitral annular calcification volume

Patient	MAC volume (mm^3^)	Septal lateral (mm)	Intercommissural (mm)	Perimeter (mm)	Area (mm^2^)
1	1687	44.1	50.8	153	1834
2	1106	36.9	31.3	108	916
3	2723	32.5	39.0	112	985
4	1816	31.6	37.0	110	932
5	1384	36.6	40.8	123	1178
6	3539	35.3	36.4	114	1027
Mean	2043 ± 916	36.2 ± 4.4	39.2 ± 6.5	120 ± 17	1145 ± 350

Abbreviation: MAC, mitral annular calcification.

**Table 3:** Mitral annular calcification score∗

Subject	MAC thickness (mm)	MAC distribution (^o^)	Trigone involvement	Leaflet involvement	MAC score	MAC severity
1	8.2 (2)	117 (1)	One (1)	One (1)	5	Moderate
2	5.6 (2)	189 (2)	Both (2)	None (0)	6	Moderate
3	11.0 (3)	235 (2)	Both (2)	One (1)	8	Severe
4	10.7 (3)	206 (2)	Both (2)	One (1)	8	Severe
5	10.1 (3)	116 (1)	None (0)	One (1)	5	Moderate
6	10.3 (3)	197 (2)	One (1)	One (1)	7	Severe

*Notes.* Numbers are presented as Measurement (Score) as defined by Guerrero et al.

Abbreviation: MAC, mitral annular calcification.

∗ Guerrero M, et al. JACC Cardiovasc Imaging 2020; 13:1945–57.

### Procedural and In-hospital Outcomes

Procedural and in-hospital outcomes are described in Table 4. In brief, technical success was achieved in all patients (100%) with a clinically significant reduction in MR. At discharge, 4/5 (80%) patients had no PVL or trace PVL, and 1/5 (20%) had mild MR. The postprocedural mitral valve mean gradient was 2.1 ± 0.9 mm Hg. One of the three patients implanted via transseptal access required closure of the atrial septal defect. There was no intraprocedural mortality, device malposition, or embolization. One patient expired 3 days postprocedure due to complications related to the transapical access. All other patients were discharged from the hospital without issues, and long-term clinical outcomes were monitored.

**Table 4:** Procedural and in-hospital outcomes

Parameter	Metric
Delivery system	
Transapical (34F)	50% (3)
Transseptal (29F)	50% (3)
Annular ring diameter	
40 mm	50% (3)
46 mm	33% (2)
54 mm	17% (1)
Balloon predilation or valvuloplasty	0% (0)
Device time, min	
Transapical	32 ± 16
Transseptal	41 ± 8
Balloon postdilation	0% (0)
Atrial septum closure (transseptal cases)	33% (1/3)
Procedural and device results	
Technical success	100% (6)
In-hospital mortality	17% (1)
Cardiovascular related (transapical)	17% (1)
Noncardiovascular	0% (0)
LVOT obstruction	0% (0)
Device thrombosis	0% (0)
Device embolization	0% (0)
Device malposition	0% (0)
Device retrieval	0% (0)
Stroke or transient ischemic attack	0% (0)
Myocardial infarction	17% (1)
New pacemaker implantation	0% (0)
Conversion to open heart surgery	0% (0)
Major vascular complications	0% (0)
Echocardiographic outcomes at discharge	
Residual mitral regurgitation and/or PVL	
None/trace	80% (4)
1+	20% (1)
2+ or greater	0% (0)
Mitral mean gradient (postimplant), mm Hg	2.1 ± 0.9

*Notes.* Values are presented as mean ± SD or % (n).

Abbreviation: PVL, paravalvular leak.

### Thirty-day and Long-term Clinical Outcomes

**Figure 3:**
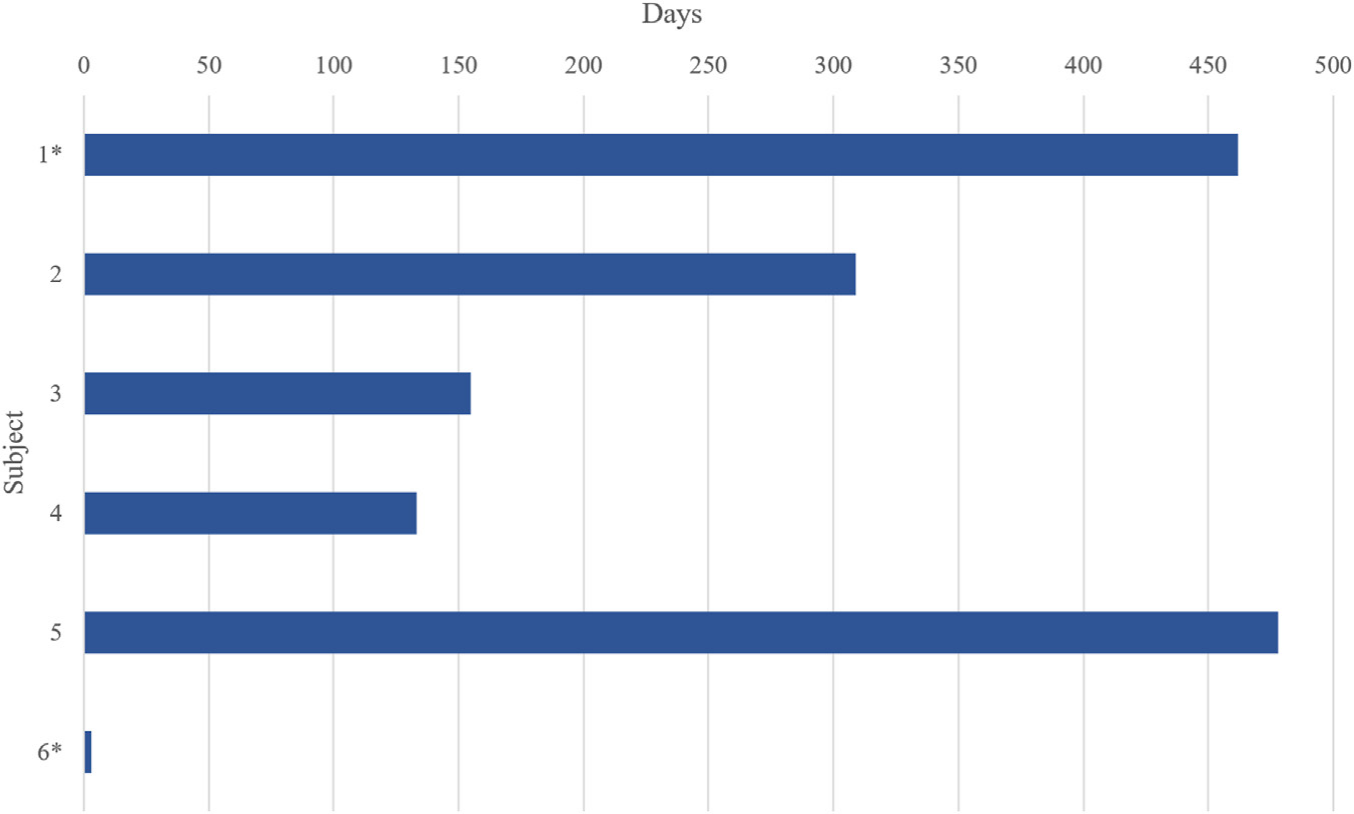
**Postimplant long-term survival of patients with mitral annular calcification treated using the AltaValve in the study.** ∗Expired subjects.

Patients were followed for a median of 220 days (range 3–466 days) postprocedure (Figure 3). The longest follow-up was 462 days postimplant. One in-hospital death, related to transapical access and management, was reported prior to 30 days. There were no additional mortality reports at 30 days and at 3-month follow-up. Two patients, both implanted transapically, completed a 1-year follow-up. One patient expired at 462 days postprocedure due to progression of heart failure unrelated to the study device. No major device-related adverse events were reported for the patients in follow-up. There have been no reports of LVOT obstruction, valve thrombosis, hemolysis, new pacemaker implantation, and/or embolization of the prosthesis.

**Figure 4:**
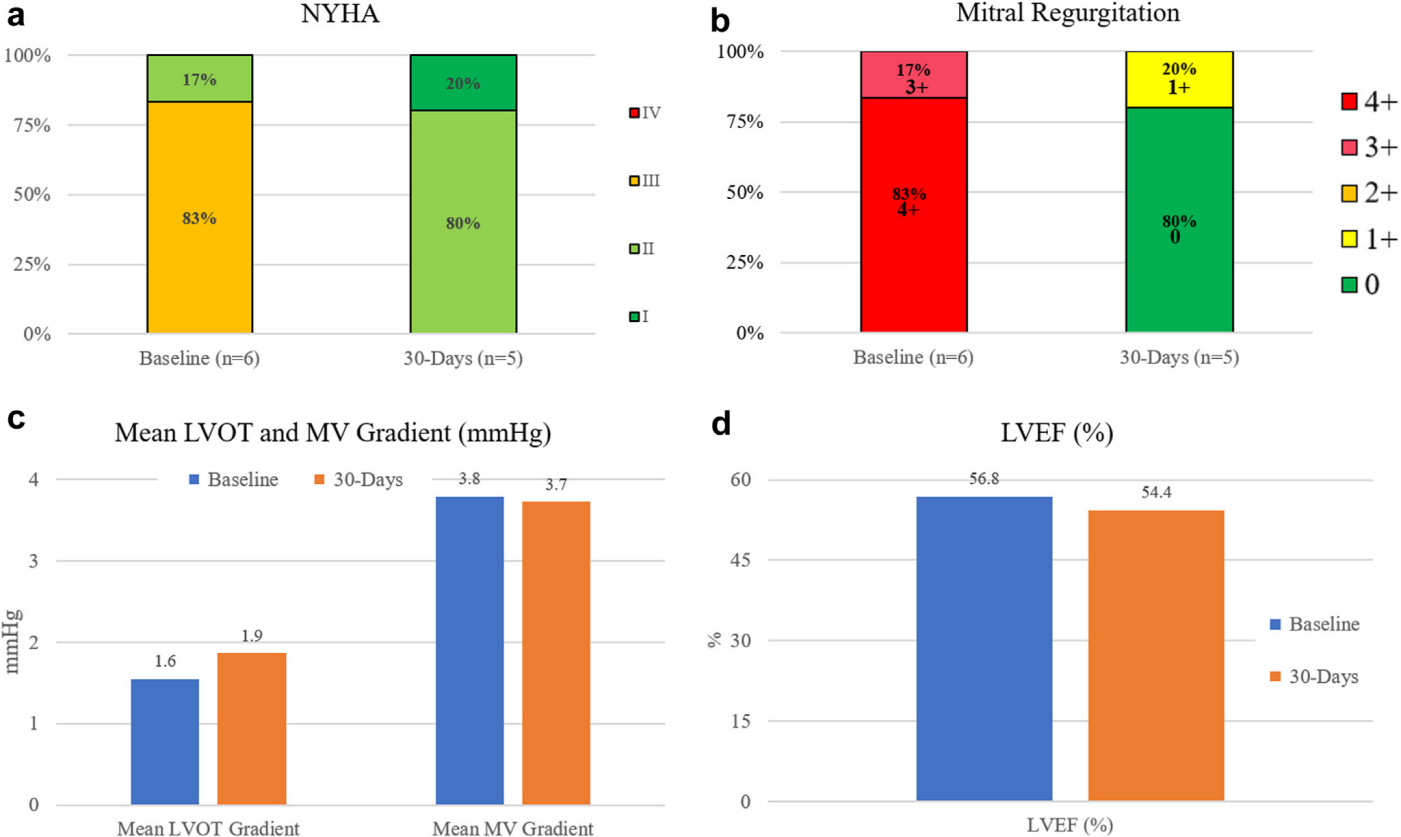
**Clinical outcomes at baseline and 30-days postimplant.** Comparison of (a) New York Heart Association (NYHA) functional class; (b) mitral regurgitation; (c) mean left ventricular outflow tract (LVOT) gradient and mitral valve (MV) gradient; and (d) left ventricular ejection fraction (LVEF) at baseline and 30 days.

At 30 days, all patients (100%) were in NYHA class I/II, showing marked improvement compared with baseline. Transthoracic echocardiography assessments showed 1 (20%) patient with mild MR at 30-day follow-up. Valve hemodynamics and performance show a mean mitral valve gradient of 3.7 ± 1.4 mm Hg. The LVOT gradients were measured at an average of 1.9 mm Hg as compared with 1.6 mm Hg measured postimplantation. No significant changes in LV function were observed during follow-up (Figure 4).

### Postimplant CT Assessments

**Figure 5:**
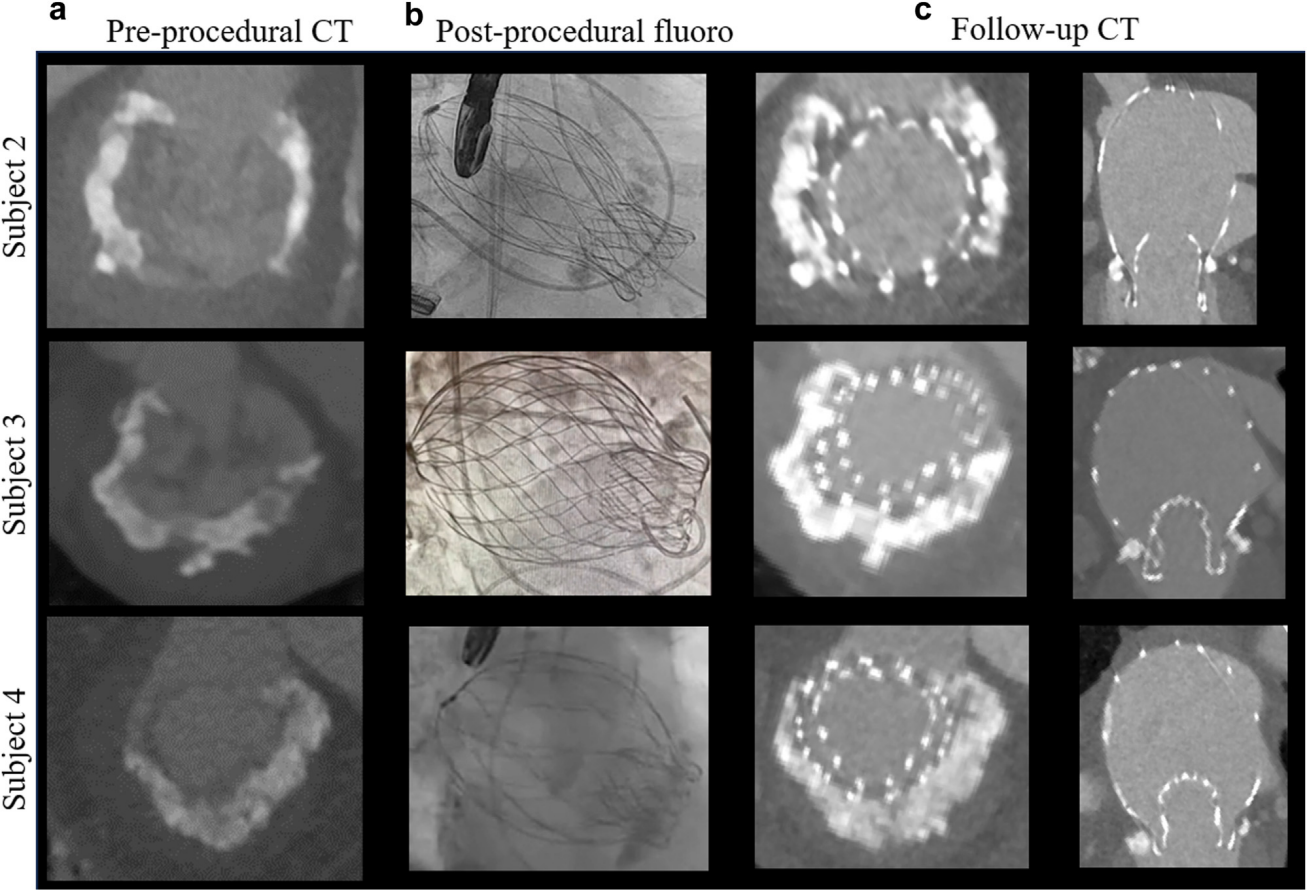
**Postimplantation imaging of patients with severe mitral annular calcification treated with the AltaValve.** Images of 3 patients with severe mitral annular calcification (MAC) treated with the AltaValve. (a) Preprocedural electrocardiogram (ECG)-gated, contrast-enhanced computed tomography (CT) images depicting the MAC; (b) postprocedural fluoroscopic images showing the fully deployed AltaValve; (c) follow-up CT images demonstrating the interaction between the AltaValve bioprosthesis and the MAC.

Postimplant CT was obtained in 5 patients at 30 days or greater to assess the interaction of the prosthesis with the MAC. Representative examples from three patients are shown in Figure 5. All three patients were screen-failed from at least 1 TMVR trial due to MAC and/or risk of LVOT obstruction. The CT images show the AltaValve with good apposition to the left atrium (Figure 5, Panel c). The cross-sectional images show that the implant conforms to the MAC. There was no evidence of leaflet or implant thrombosis from the CT assessments. Despite LVOT being at-risk for obstruction, no obstruction was noted. Additionally, the LVOT area increased in systole as compared with diastole for all patients.

## Discussion

The current study demonstrated the feasibility of successfully treating patients presenting with severe MR and significant MAC using the AltaValve atrial fixation TMVR technology. The ability to safely position the implant with sustained resolution of MR and no device-related mortality was achieved in all patients.

The technical challenges of treating patients with severe MAC using different TMVR technologies have been well reported. These challenges resulted in reduced technical success as compared with non-MAC patients. In the MAC Global Registry, Guerrero et al. reported a technical success rate of 72% in 64 patients when using balloon-expandable valves.^13^ In the prospective MITRAL trial, Guerrero et al. reported technical success of 74 and 80% using a transatrial approach and a transseptal approach, respectively.^14^ Similarly, Yoon et al. reported 54% technical success in 37 patients when using prosthetic valves in MAC patients using either a transapical or transseptal approach.^15^ In a smaller subset of 9 patients, Sorajja et al. reported an 89% technical success rate using the Tendyne valve (Abbott) with a transapical approach to treat patients with MAC.^16^ Comparatively, >95% technical success is generally reported for TMVR implantation in non-MAC patients. This difference highlights the technical challenges associated with MAC, pointing toward the need for technological advancements to improve procedural outcomes. In our study, we report a 100% technical success rate using both transapical and transseptal implantation approaches. Overall, procedure times for both approaches were <1 ​hour, despite the early experiences with the technology at the implanting sites.

Surgical literature and off-label use of TMVR technologies to treat MAC patients clearly show an increased mortality and morbidity risk. In their review of clinical experiences using transcatheter technologies to treat MAC, Alexis et al.^9^ reported overall in-hospital, 30-day, and 1-year mortality rates of 16.7, 22.7, and 43%, respectively, using either a transapical or transseptal approach. Similarly, Guerrero et al. report a 25 and 53.7% mortality rate at 30 days and 1 year postprocedure in the MAC Global Registry^13^^,^^17^ Given the complexity of the anatomy and treatment risks, minimizing procedural invasiveness via a transseptal approach may have procedural and clinical benefits.

In our study, the patients were elderly, with an average age of 82 years, which is significantly higher when compared with patients treated in the MAC Global Registry (73 ± 13 years).^13^ Yet, we reported no procedural mortality and 17% mortality (1/6) at 30-day follow-up. Among the patients treated using the transseptal delivery system, there was no mortality, and all these patients have completed more than 4 months of follow-up (range 121–297 days). There are limited literature reports on the use of TMVR-dedicated technologies being used to treat severe MAC patients using a transseptal approach. Seshiah et al.^18^ describe a successful case report using the Intrepid valve (Medtronic) following lithotripsy of the MAC. In this context, experiences treating such patients with severe MAC—especially with a transseptal delivery system—are important given the high complication rate for patients with high or prohibitive surgical risk.

It has been widely reported that dynamic LVOT obstruction after treatment of patients with MAC requires reintervention and impacts survival. Yoon et al. showed a significant difference in the incidence of procedural mortality, conversion to surgery, reintervention to treat, and technical success in patients with or without LVOT obstruction^15^ Guerrero et al. found that LVOT obstruction was a strong independent predictor of 30-day and 1-year mortality.^17^ In these 2 studies, postimplant LVOT obstruction was reported in 40 and 11% of the cases, respectively. Several screening strategies to minimize LVOT obstruction have been developed and reported.^15^^,^^19^, ^20^, ^21^ LVOT obstruction risk includes both anatomical and device-specific factors. Anatomic risk factors include a small LVOT at baseline, long anterior leaflet length, small LV volume in end systole, prominent septal hypertrophy, and aorta mitral angle (>55^o^). While the understanding of anatomical features that impact LVOT obstruction is useful for patient screening, device-specific interactions that impact long-term risks of obstruction are being investigated. In this regard, AltaValve may represent an attractive treatment option for patients presenting with MAC who are at risk for LVOT obstruction.

The majority of TMVR technologies rely on subvalvular anchoring—either to the native mitral annulus, leaflets, or LV using rigid anchoring mechanisms. It is understandable to use rigid anchoring methods to prevent device migration or embolization, but this impacts and reduces native annular dynamics. Normal cardiac motion expands the LVOT during ventricular systole to facilitate blood flow to the body. Subvalvular anchoring, especially in MAC patients, further impacts the annular dynamics and increases the risk of LVOT obstruction. In this context, atrial fixation of the AltaValve preserves the dynamics of the native mitral annulus, thus allowing the device to compress during ventricular systole. This suggests that the lack of rigid anchoring within the mitral annulus or LV may be beneficial in treating such patients with small LVOTs both procedurally as well as long term. All patients treated in this study were screen-failed for at least 1 other technology due to the risk of LVOT obstruction. Follow-up images obtained 30 days or greater postimplant show patent LVOT in all patients with an average LVOT gradient <3 mm Hg in each. The cross-sectional images clearly show the compression of the annular ring anteriorly, resulting in an expansion of the LVOT area during ventricular systole compared with diastole. The implications of this finding are critical since it decreases the risk of LVOT obstruction, thus expanding the pool of potentially treatable patients with or without MAC.

### Study Limitations

This study includes a small number of patients and is limited to experiences in patients with severe MAC either as part of an ongoing EFS or via compassionate use treatment. Clinical studies are ongoing to evaluate the technology’s performance in non-MAC patients globally. The clinical events reported for the patients in the EFS are adjudicated by an independent panel of physicians, who constitute the clinical events committee. The clinical events for the compassionate use patient are site reported. Further clinical investigation in a larger cohort of MAC patients is warranted to refine patient selection as well as evaluate long-term safety and effectiveness of the technology.

## Conclusions

The current study presents six patients with severe MR and severe MAC who were successfully treated with the AltaValve TMVR technology. The atrial fixation of the technology minimizes the risk of LVOT obstruction and potentially expands treatable patients, especially with MAC. A larger multicenter investigation is warranted to evaluate the safety and effectiveness of the technology.

## Ethics Statement

All patients were treated either on a compassionate use basis (n = 1) or enrolled as part of the AltaValve Early Feasibility Study (NCT03997305) (n = 5). For the compassionate use patient, appropriate hospital ethics committee and regulatory approvals were obtained prior to the case (ANSM, France). Informed consent was obtained from all patients prior to the procedure.

## Funding

The study was sponsored by 4C Medical Technologies, Maple Grove, MN.

## Disclosure Statement

P. Généreux has been a consultant for 4C Medical, Abbott Vascular, Abiomed, Boston Scientific, CARANX Medical, Cardiovascular System Inc, Edwards Lifesciences, GE Healthcare, iRhythm Technologies, Medtronic, Opsens, Pi-Cardia, Puzzle Medical, Saranas, Shockwave, Soundbite Medical Inc, and Teleflex; has been an advisor to Abbott Vascular, Abiomed, BioTrace Medical, Edwards Lifesciences, and Medtronic; has received speaker fees from Abbott Vascular, Abiomed, BioTrace Medical, Medtronic, Shockwave, and Siemens; has been a principal investigator of 4C Medical for a feasibility study, Cardiovascular System Inc for Eclipse Trial, and Edwards Lifesciences for EARLY-TAVR and PROGRESS trials; holds equity in Pi-Cardia, Puzzle Medical, Saranas, and Soundbite Medical Inc; and has been a proctor for and received institutional grants from Edwards Lifesciences. Krzysztof Wróbel is a consultant and proctor for 4C Medical. M. J. Rinaldi is a consultant for Abbott Vascular, a consultant for Edwards Lifesciences, a consultant for Bostin Scientific, a consultant for Johnson and Johnson, a consultant for Abiomed, and a consultant for Shockwave Medical. T. Modine served on the advisory board of Medtronic, Valfix, and Valcare, is a consultant for Abbott, Edwards Lifesciences, Microport, GE, and Boston Scientific, received research grants from Edwards Lifesciences, Medtronic, and Abbott, and is the principal investigator of Apollo trial, Trinity study, and Neocord study. V. Bapat is a consultant for Edwards Lifesciences, Medtronic, Abbott, Boston Scientific, and Anteris. V. Ninios is a proctor for 4C Medical, Abbott, and Boston Scientific. P. Sorajja served on the advisory boards of 4C Medical, Abbott Structural (Cephea, Global Valve Masters, Global Complexity Score, Tricuspid Advisory Board), Boston Scientific SHV Strategic Advisory Board, Boston Scientific Advisory Board, Medtronic Structural Advisory Board, and VDyne Advisory Board. Serves as a consultant for 4C Medical, Abbott Structural, Adona, Boston Scientific, Edwards Lifesciences, Foldax, GE Medical, GLG, Medtronic, Phillips, Siemens, WL Gore, vDyne, xDot. Serves as National or Global Principal Investigator of TRILUMINATE Pivotal US Trial, SUMMIT MAC Pivotal Trial, EXPAND 2 Pivotal US Trial, HighLife (USA) EFS, and VDyne EFS.
